# Impact of PET/CT system, reconstruction protocol, data analysis method, and repositioning on PET/CT precision: An experimental evaluation using an oncology and brain phantom

**DOI:** 10.1002/mp.12623

**Published:** 2017-11-19

**Authors:** Syahir Mansor, Elisabeth Pfaehler, Dennis Heijtel, Martin A. Lodge, Ronald Boellaard, Maqsood Yaqub

**Affiliations:** ^1^ Department of Radiology & Nuclear Medicine VU University Medical Center De Boelelaan 1117, 1081 HV Amsterdam The Netherlands; ^2^ Faculty of Medical Sciences Nuclear Medicine and Molecular Imaging Hanzeplein 1, 9713 GZ Groningen The Netherlands; ^3^ PET Center Johns Hopkins Hospital Nelson B1125, 1800 Orleans Street Baltimore MD 21287 USA

**Keywords:** 3D Hoffman brain phantom, IQ NEMA phantom, PET/CT, phantom repositioning, repeatability, reproducibility

## Abstract

**Purpose:**

In longitudinal oncological and brain PET/CT studies, it is important to understand the repeatability of quantitative PET metrics in order to assess change in tracer uptake. The present studies were performed in order to assess precision as function of PET/CT system, reconstruction protocol, analysis method, scan duration (or image noise), and repositioning in the field of view.

**Methods:**

Multiple (repeated) scans have been performed using a NEMA image quality (IQ) phantom and a 3D Hoffman brain phantom filled with ^18^F solutions on two systems. Studies were performed with and without randomly (< 2 cm) repositioning the phantom and all scans (12 replicates for IQ phantom and 10 replicates for Hoffman brain phantom) were performed at equal count statistics. For the NEMA IQ phantom, we studied the recovery coefficients (RC) of the maximum (SUV_max_), peak (SUV_peak_), and mean (SUV_mean_) uptake in each sphere as a function of experimental conditions (noise level, reconstruction settings, and phantom repositioning). For the 3D Hoffman phantom, the mean activity concentration was determined within several volumes of interest and activity recovery and its precision was studied as function of experimental conditions.

**Results:**

The impact of phantom repositioning on RC precision was mainly seen on the Philips Ingenuity PET/CT, especially in the case of smaller spheres (< 17 mm diameter, *P *< 0.05). This effect was much smaller for the Siemens Biograph system. When exploring SUV_max_, SUV_peak_, or SUV_mean_ of the spheres in the NEMA IQ phantom, it was observed that precision depended on phantom repositioning, reconstruction algorithm, and scan duration, with SUV_max_ being most and SUV_peak_ least sensitive to phantom repositioning. For the brain phantom, regional averaged SUVs were only minimally affected by phantom repositioning (< 2 cm).

**Conclusion:**

The precision of quantitative PET metrics depends on the combination of reconstruction protocol, data analysis methods and scan duration (scan statistics). Moreover, precision was also affected by phantom repositioning but its impact depended on the data analysis method in combination with the reconstructed voxel size (tissue fraction effect). This study suggests that for oncological PET studies the use of SUV_peak_ may be preferred over SUV_max_ because SUV_peak_ is less sensitive to patient repositioning/tumor sampling.

## 1 Introduction

[^18^F]Fluorodeoxyglucose (^18^F‐FDG) positron emission tomography and computed tomography (PET/CT) is being used for staging and tumor response assessment in oncology.^1^, ^2^, ^3^, ^4^, ^5^, ^6^, ^7^ The analysis of [^18^F]‐FDG^8^ uptake in tumors can be performed semiquantitatively using the standard uptake value (SUV) rather than using visual assessment of relative change. The main drawback of using SUV is its sensitivity to various technical factors, such as image reconstruction settings^9^ and segmentation strategies.^10^, ^11^, ^12^ The impact of different image acquisition and processing methods on SUV are well understood and to mitigate these effects,^13^ various standardization efforts are made, especially in multicenter clinical trials. In order to yield a high reproducibility, standard operating procedures (SOPs) or guidelines need to be followed that address patient preparation, image acquisition and processing, and data analysis and interpretation. For longitudinal studies, i.e., when quantitatively measuring tumor response to therapy, it is important to understand the precision of the quantitative metric being used to measure change in tracer uptake. Several studies have reported^14^, ^15^, ^16^ repeatabilities ranging from 10% to 15%, on average. This precision arises from several clinical and technical contributions, such as uncertainties in administered activity, variability in patient preparation and physiological condition (blood glucose level) *et cetera*, and also from image noise due to variability in scan statistics. Very few FDG SUV precision studies report within‐patient coefficients of variation less than 10% and it is unclear if this is limited by technical as opposed to patient‐related factors. Technical limitations have partly been assessed using phantoms filled with long half‐life isotopes and reassessed at multiple PET centers.^17^ However, these effects were not yet assessed for brain protocols using an anthropomorphic brain phantom. In addition, new reconstruction algorithms have been developed for clinical PET/CT systems, incorporating the system point spread function, that are able to improve spatial resolution.

The aim of this study was, therefore, to experimentally evaluate PET/CT precision dependence on reconstruction protocol, scan duration, and image analysis methods. Most importantly we compared to what extent precision of various quantitative uptake metrics obtained with different reconstruction protocols, voxel sizes, and scan durations depend on phantom repositioning versus static placement of the phantom. These studies were performed for both an oncology and brain phantom. In most experimental studies reported to date the repositioning aspect was not included. As partial volume effects are in part caused by the so‐called tissue fraction effect (voxel size), the actual ‘voxel sampling’ of small objects may be an additional source of uncertainty. In clinical longitudinal studies, patients are not repositioned in exactly the same manner during all scans. Therefore, it is important to assess these repositioning effects and to determine which of the analysis methods can mitigate these effects best. To this end, PET phantoms for quantitative performance assessment were scanned on two different PET/CT systems. The acquisitions were repeated (n = 12 for IQ phantom and n = 10 for Hoffman brain phantom) with and without phantom repositioning, while keeping count statistics equivalent between replicates. Additionally, the acquired data were reconstructed using various clinically applied reconstruction protocols and frame durations. All data were analyzed with common quantitative metrics, such as SUV_max_, SUV_peak_, or SUV_mean_.

## 2 Materials and methods

### 2.A. Phantom experiments

Phantom experiments were performed on an Ingenuity PET/CT scanner (Philips Healthcare, Cleveland, OH, USA) and the Biograph mCT 40 (Siemens Healthcare, Knoxville, TN, USA). All emission data were reconstructed using the vendor‐provided time of flight iterative reconstruction method including all corrections needed for quantification such as scatter, random, normalization, and attenuation correction. The Philips Ingenuity system uses an iterative reconstruction algorithm (BLOB‐OS‐TF) with 3 iterations and 33 subsets, and the Siemens Biograph system uses a 3D iterative reconstruction algorithm (OSEM) with 3 iterations and 21 subsets. For both systems, a low‐dose CT, using vendor recommended settings, was used for attenuation correction.

Moreover images were generated with and without point spread function (PSF) or resolution modeling. For the Philips Ingenuity PET/CT system, the resolution modeling is implemented as a postreconstruction iterative deconvolution method (and used with the vendor provided default settings). The Philips Ingenuity system reconstructs images with a voxel size of either 4 × 4 × 4 mm^3^ or 2 × 2 × 2 mm^3^ with a corresponding matrix of 144 × 144 × 45 or 288 × 288 × 90 for body mode acquisitions. Brain mode acquisitions yield images with a voxel size of 2 × 2 × 2 mm^3^ and a matrix of 128 × 128 × 90 (applied only in case of the 3D Hoffman phantom, as discussed below). Resolution modeling on the Siemens Biograph system is implemented within the reconstruction process, i.e., included in the system matrix. Data collected on the Siemens Biograph PET/CT system are reconstructed with a voxel size of either 3.1819 × 3.1819 × 2 mm^3^ or 2.0364 × 2.0364 × 2 mm^3^ with a corresponding matrix of 256 × 256 × 111 or 400 × 400 × 111 for body mode acquisitions. Brain mode acquisitions are reconstructed with a voxel size of 2.0364 × 2.0364 × 2 mm^3^ and matrix of 400 × 400 × 111.

Two different phantoms were evaluated. First, the NEMA NU‐2 Image Quality (IQ) phantom (Data Spectrum, Hillsborough, NC, USA) was used. This phantom is well known for its use in NEMA NU‐2 IQ PET performance measurements and for its use in standardization of multicenter PET studies (EANM‐EARL).^18^ The phantom consists of a large background volume (9400 mL) with six spheres with inner diameters of 10, 13, 17, 22, 28, and 37 mm. The spheres and the background were filled with an ^18^F solution following EANM/EARL recommendations and resulted in sphere/background ratios of approximately 10:1. The ‘true’ activity concentration in the phantom was derived from the activity measurement with a dose‐calibrator and the known volume of the phantoms. Moreover, activity concentrations were cross‐checked by measuring samples on a gamma well counter. Two series of scans were performed for each PET/CT system. First, the IQ phantom was filled once (ranging from 1.75 to 3.08 kBq ml^−1^ in the background compartment and 17.78 to 28.63 kBq ml^−1^ in the spheres) and scanned in one fixed position for 120 min. Data were reconstructed in 12 frames at three different frame durations (2, 4, and 5 min for the first reconstructed frame). In order to keep scan statistics constant between all reconstructed images, frame duration was increased for each subsequent reconstructed frame to compensate for radioactive decay (i.e., yielding similar count statistics for each subsequent frame). Secondly, the IQ phantom was filled once and rescanned (both low‐dose CT and PET) 12 times while randomly repositioning the phantom at different angles along any axis (< 5 degrees) and translations (x, y, z), resulting in displacements of up to 20 mm. Each of the acquisitions was reconstructed with frame durations to yield the same count statistics as achieved with the first set of (stationary phantom) measurements.

Secondly, we acquired data for the 3D Hoffman brain phantom (Data Spectrum, Hillsborough, NC, USA). Similar to the IQ phantom experiment, the phantom was scanned in two series for each PET/CT system: one using the same phantom position over 120 min (activities ranging from 59.69 to 125 MBq in the phantom at start scanning) and a series consisting of rescanning at 10 different phantom repositions (activities ranging from 62.34 to 114 MBq in the phantom at start scanning). Similar to the IQ phantom studies, data were reconstructed with three different frame durations (2, 4, and 5 min for the first frame). The frame duration was again increased to compensate for radioactive decay (i.e., yielding similar count statistics for each frame).

### 2.B. Regional assessments

Regional assessment of the experiments was performed using several automated (IQ Phantom) and manual image segmentation methods (3D Hoffman phantom). Automated segmentation of the spheres of the IQ phantom was performed using the EARL analysis tool which generated volumes with background corrected isocontours set at 50% of SUV_max._ 18 From these delineations, we derived the maximum (SUV_max_), peak (SUV_peak_), and mean (SUV_mean_) uptake in each of the images. The peak SUV was derived from a 1 ml spherical volume of interest (VOI) positioned to yield the highest average VOI value across the lesion (or sphere in case of the phantom). Note that the VOI analysis was performed on the original images without image registration to resemble clinical conditions as closely as possible. Next, we derived the recovery coefficient (RC_max_, RC_peak_, and RC_mean_) by dividing observed max, peak, and mean values by the expected activity concentrations. RCs were derived for each sphere and for all acquired and reconstructed emission images. RCs precision as a function of sphere size, data analysis method (max, peak, and mean), and reconstruction methods for both stationary and repositioning phantom experiments was evaluated.

**Figure 1:**
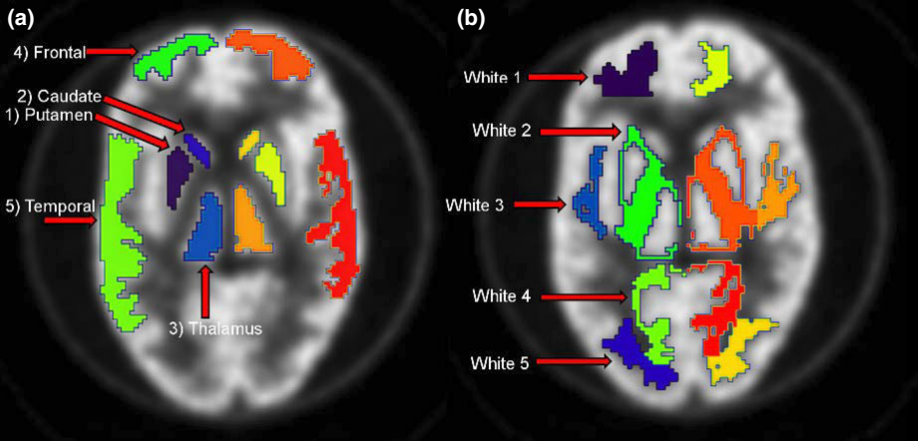
Illustration of VOIs in gray matter for both brain hemispheres in (A) and VOIs in white matter in (B). These VOIs were used to assess RC for different brain structures and regions as function of experimental condition. [Color figure can be viewed at wileyonlinelibrary.com]

For the 3D Hoffman brain phantom, several volumes of interest (VOIs) were drawn manually using a coregistered binary mask of gray and white matter of the phantom. For each hemisphere in total, five different VOIs for gray and five VOIs for white matter of different sizes were drawn as shown in Fig. 1. VOI were chosen to obtain activity concentration estimates for both cortical and more deeply located brain structures. From these VOIs, we derived the mean regional activity concentration and compared these with the actual activity concentration of the solution used to fill the phantom to produce the RC_mean_. For the repositioned phantom study, this VOI template was rigidly realigned onto the original phantom images.

## 3 Results

### 3.A. NEMA IQ phantom

**Figure 2:**
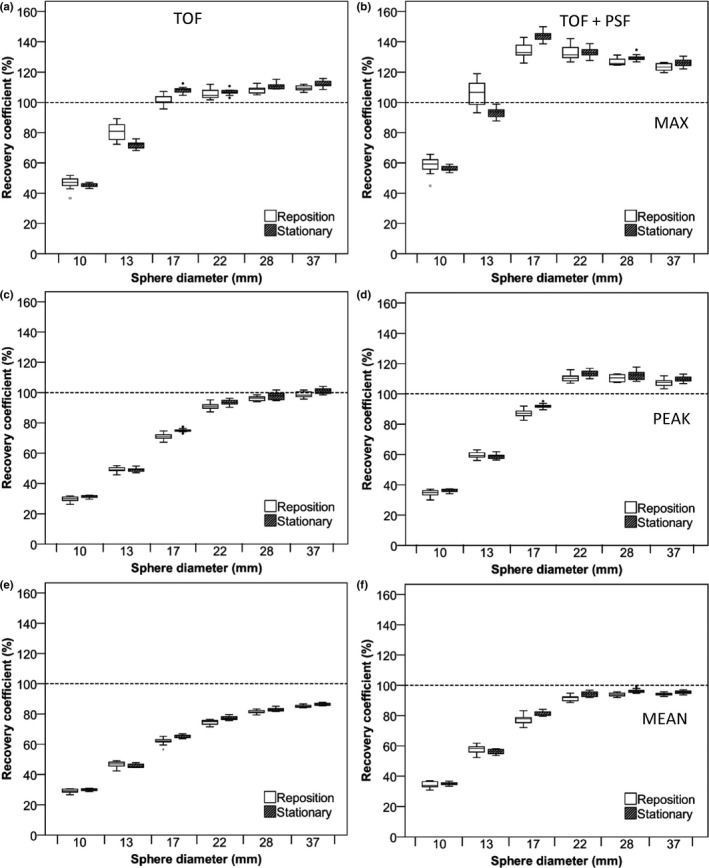
RC of NEMA IQ phantom data as a function of sphere diameter. Data were acquired on the Philips Ingenuity system and based on images with a 4 × 4 × 4 mm^3^ voxel size and 5‐min starting frame duration using TOF on the left column and TOF + PSF on the right column. Figures (A and B) represent RC (%) for max, (C and D), peak, and (E and F) mean SUVs. Dotted lines correspond to the true RC based on the true activity within the phantom spheres. Boxes represent standard deviation (SD), whiskers show ranges, and solid line depicts median of the data.

**Figure 3:**
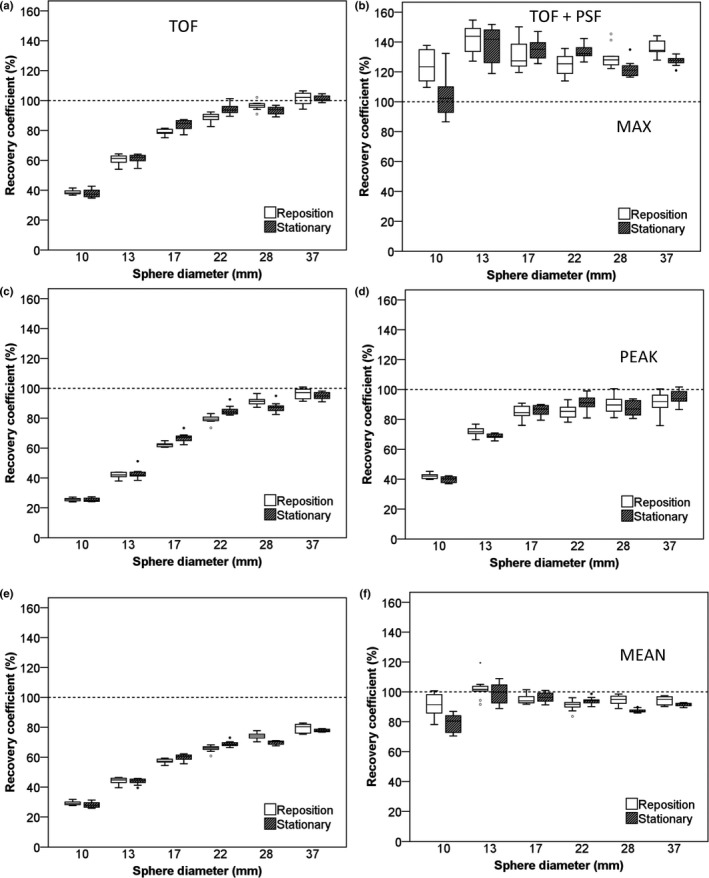
RC of NEMA IQ phantom data as a function of sphere diameter. Data were acquired on the Siemens Biograph system and based on images with a 3.1819 × 3.1819 × 2 mm voxel size and 5‐min starting frame duration using TOF on the left column and TOF + PSF on the right column. Figures (A and B) represent RC (%) for max, (C and D), peak, and (E and F) mean SUVs. Dotted lines correspond to the true RC based on the true activity within the phantom spheres. Boxes represent standard deviation (SD), whiskers show ranges, and solid line depicts median of the data.

Figures 2 and 3 illustrate recovery coefficients for the IQ phantom for images with 5‐min scan duration. In general, repositioning of the phantom increased variability of RC data compared with the stationary phantom data especially for the Philips Ingenuity system (Table 1). The additional variability due to repositioning was larger when using RC_max_ and/or using reconstructions that include PSF. Also, for both systems, use of TOF + PSF produced higher recoveries than TOF reconstruction alone and this effect (> 5% increase) was largest for RC_max_ observed with the Siemens Biograph system [Fig. 3(b)]. The PSF implementation on the Siemens Biograph also affects the smaller spheres more as compared to the implementation on the Philips Ingenuity system, which resulted in an increased RC and also strongly increased variability [Figs. 2(b), 2(f), 3(b) and 3(f)]. In supporting information Figs. S1 and S2, recovery coefficients observed for the IQ phantom for images with 2‐min scan duration are shown. Although these RCs showed somewhat larger variability, as expected due to the lower count statistics as compared to 5‐min data, overall trends were similar to those of the 5‐min data.

**Table 1:** Significant *P* values (not corrected for multiple comparisons) calculated by performing *F*‐tests between repositioned and stationary phantom datasets for different analysis and reconstruction methods and for each sphere and for 5‐min scan duration data with 4 × 4 × 4 mm^3^ voxel sizes for the Philips Ingenuity system and 3.1819 × 3.1819 × 2 mm^3^ voxel sizes for the Siemens Biograph system. Nonsignificant values are indicated with – for clarity reasons

	Sphere diameter (mm)	TOF (SUV_max_)	TOF + PSF (SUV_max_)	TOF (SUV_peak_)	TOF + PSF (SUV_peak_)	TOF (SUV_mean_)	TOF + PSF (SUV_mean_)
Philips ingenuity	10	0.001	0.001	0.001	0.010	0.053	0.013
	13	0.002	0.002	–	–	0.036	0.034
	17	0.018	4.5 × 10^−8^	0.002	1.2 × 10^−8^	0.003	4.08 × 10^−8^
	22	–	–	–	–	–	–
	28	–	–	–	–	–	–
	37	–	–	–	–	–	–
Siemens biograph mCT40	10	–	0.001	–	0.006	–	0.01
	13	–	–	–	0.009	–	–
	17	0.001	–	0.001	–	0.003	–
	22	0.001	0.040	0.004	0.030	0.002	0.032
	28	0.013	–	0.003	–	0.001	0.001
	37	–	0.001	–	–	–	–

**Figure 4:**
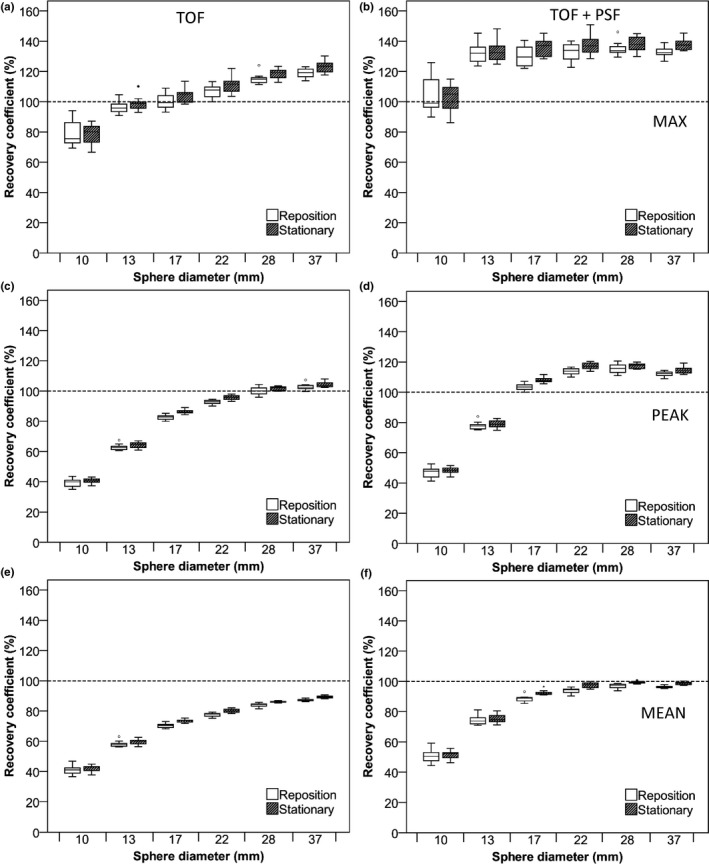
RC of NEMA IQ phantom data as a function of sphere diameter. Data were acquired on the Philips Ingenuity system and based on images with a 2 × 2 × 2 mm^3^ voxel size and 5‐min starting frame duration using TOF on the left column and TOF + PSF on the right column. Figures (A and B) represent RC (%) for max, (C and D), peak, and (E and F) mean SUVs. Dotted lines correspond to the true RC based on the true activity within the phantom spheres. Boxes represent standard deviation (SD), whiskers show ranges, and solid line depicts median of the data.

**Figure 5:**
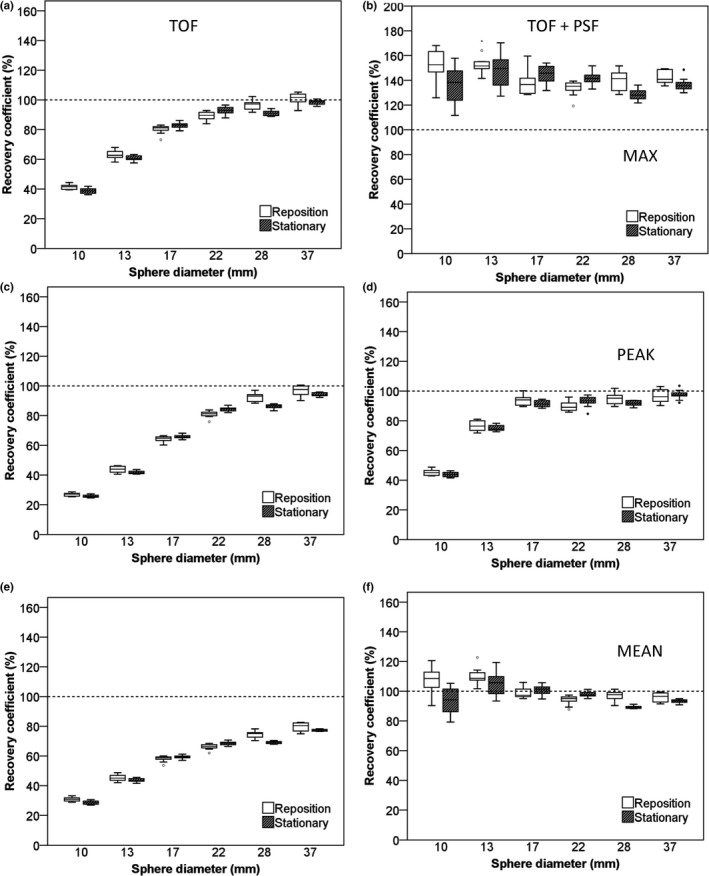
RC of NEMA IQ phantom data as a function of sphere diameter. Data were acquired on the Siemens Biograph system and based on images with a 2 × 2 × 2 mm^3^ voxel size and 5‐min starting frame duration using TOF on the left column and TOF + PSF on the right column. Figures (A and B) represent RC (%) for max, (C and D), peak, and (E and F) mean SUVs. Dotted lines correspond to the true RC based on the true activity within the phantom spheres. Boxes represent standard deviation (SD), whiskers show ranges, and solid line depicts median of the data.

Recovery coefficients for images reconstructed with smaller voxel sizes (2 × 2 × 2 mm^3^) are shown in Figs. 4 and 5 (5‐min scan duration) and supporting information Figs. S3 and S4 (2‐min scan duration). Comparing the differences between Fig. 2 and 4 and between Fig. 3 and 5 showed that smaller voxel sizes result in increased variability in the observed recoveries. This effect is larger for the Philips Ingenuity than for the Siemens Biograph system. For both scanners, the variability of RC was now comparable between repositioning and stationary phantom experiments (Table 2). Moreover, shorter frame durations increased variability in the observed recoveries. In general, RC_max_ was more sensitive to noise and phantom repositioning than the other quantitative metrics. Tables 1 and 2 summarize the *F*‐test significance (not corrected for multiple comparisons), for differences in precision between the stationary scan and repositioning phantom data for the various analysis methods and voxel sizes.

**Table 2:** Significant *P* values (not corrected for multiple comparisons) calculated by performing *F*‐tests between repositioned and stationary phantom datasets for different analysis and reconstruction methods and for each sphere and for 5‐min scan duration data with 2 × 2 × 2 mm^3^ voxel sizes for both the Philips Ingenuity system and the Siemens Biograph system. Nonsignificant values are indicated with – for clarity reasons

	Sphere diameter (mm)	TOF (SUV_max_)	TOF + PSF (SUV_max_)	TOF (SUV_peak_)	TOF + PSF (SUV_peak_)	TOF (SUV_mean_)	TOF + PSF (SUV_mean_)
Philips ingenuity	10	0.006	–	–	–	–	–
	13	0.005	–	–	–	–	–
	17	0.010	–	–	–	–	0.002
	22	0.001	–	–	–	–	–
	28	–	–	0.021	0.028	0.003	0.009
	37	0.016	–	–	–	–	–
Siemens biograph mCT40	10	0.046	0.011	0.025	–	0.032	0.020
	13	–	–	0.008	–	–	–
	17	0.024	–	–	–	–	–
	22	0.001	0.004	0.001	0.027	0.001	0.004
	28	0.007	0.001	0.003	0.047	0.000	0.001
	37	–	0.011	–	–	–	0.021

### 3.B. 3D Hoffman brain phantom evaluation

**Figure 6:**
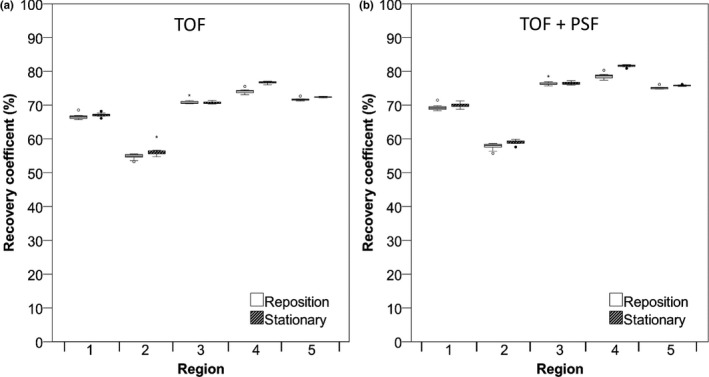
RC (%) of Hoffman phantom data in different gray matter regions. Data were acquired on the Philips Ingenuity system and reconstructed using TOF (A) and TOF + PSF (B). RC for 5‐min frame duration are shown. Boxes represent standard deviation (SD), whiskers show ranges, and solid line depicts median of the data.

**Figure 7:**
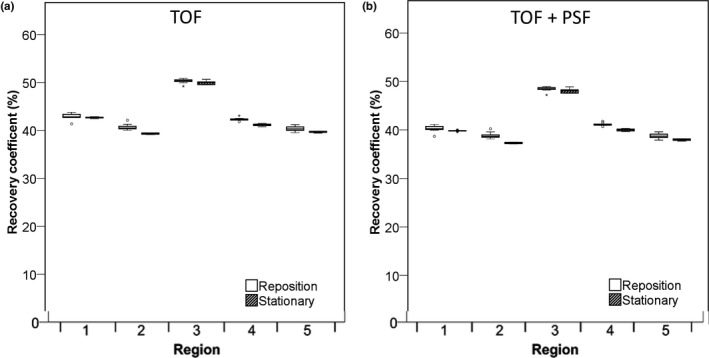
RC (%) of Hoffman phantom for different white matter regions. Data were acquired on the Philips Ingenuity system and reconstructed using TOF are shown (A) and with TOF + PSF in (B). Data for 5‐min frame durations are shown. Boxes represent standard deviation (SD), whiskers show ranges, and solid line depicts median of the data depicts median of the data.

Box plots in Fig. 6 demonstrate the RC_mean_ for several gray matter regions drawn in the Hoffman brain phantom acquired on the Philips Ingenuity system. There was no significant difference in RC variability between repositioned and stationary scans and when using shorter frame durations (data not shown). PSF‐based reconstructions yielded slightly higher RCs (~3%). Gray matter recoveries were similar, but slightly more variable for the repositioning data, on the Siemens Biograph system (data not shown). Figure 7 shows RC_mean_ for the white matter regions acquired on the Philips Ingenuity system. For white matter, the Philips Ingenuity system showed ~10% lower values than the Siemens Biograph system. In addition, for the Philips Ingenuity system with PSF reconstruction, the RC values were slightly lower than those obtained without PSF in white matter regions, while the Siemens Biograph system yielded similar results for the reconstructions with and without PSF.

## 4 Discussion

### 4.A. NEMA IQ phantom

The impact of phantom repositioning on RC precision can clearly be seen in Figs. 2 and 3 (and supporting information Figs. S1 and S2), especially in the case of smaller spheres (< 17 mm diameter, Table 1, *P* < 0.05) for all analysis methods used. However, this effect was smaller for the Siemens Biograph system, possibly because of the smaller voxel sizes in body mode imaging (20.2 mm^3^) compared with those used on the Philips Ingenuity system (64.0 mm^3^). This finding is supported by reconstructions with smaller voxel sizes for both systems showing that phantom repositioning has a smaller impact on RC precision if smaller voxel sizes (2 × 2 × 2 = 8 mm^3^) are used (Figs. 4 and 5, and supporting information Figs. S3 and S4). Although some of the differences were statistically significant (Tables 1 and 2), the actual differences are very small and likely clinically not relevant. In case of smaller voxels (< 4 × 4 × 4 ~ 64 mm^3^), the impact of noise (due to less count per voxel) seems to have a larger effect on RC variability than that resulting from phantom repositioning. The precision is even worse when shorter scan durations are used in combination with small voxel sizes as shown in supplemental Figs. 3 and 4. For all reconstructions, use of regionally averaged values, such as in case of RC_mean_ or RC_peak_ shows less dependence on phantom repositioning than RC_max_. Moreover, it was found that particularly RC_max_ shows upward bias with decreasing scan duration or worse scan statistics, as was shown before by Boellaard et al.,^10^ Lodge et al.,^19^ and Doot et al.^17^ A possible strategy to reduce uncertainty caused by scanner differences, noise and repositioning could therefore be achieved by the use of SUV_peak_ and this method might be the method of choice for tumor imaging in a clinical setting. Our findings are in good agreement with the study by Lodge et al.^19^ suggesting that the peak value is a more robust metric, not only experimentally^20^ but also in clinical practice.^19^ Moreover, as was shown by Makris et al.,^21^ SUV_peak_ depends less on differences in image resolution and might, therefore, be an attractive method in multicentre studies. A drawback of SUV_peak_ is the lower recovery for smaller spheres/tumors when the size of the peak VOI is equal to or larger than that of the sphere/tumor such that background activity is included within the VOI. The latter explains also why for the Siemens data in Fig. 3, when using PSF during the reconstructions, RC_peak_ still show low recoveries for the smallest spheres, while much higher recoveries were seen for RC_max_ or RC_mean_. The low recoveries of SUV_peak_ for small spheres (< 12 mm diameter) may hamper its application for very small tumors and the use of SUV_peak_ in a longitudinal setting, e.g., to measure treatment response, therefore warrants further exploration.

The choice of acquisition settings and reconstruction algorithm can also heavily affect the quantitative precision. As expected, shorter scans (i.e., 2‐min scan duration) tend to provide overestimated RC_max_ which is consistent with the finding by Boellaard et al.^10^ and Akamatsu et al.^20^ Furthermore, data in this study showed an increase in RC variability from 20 to 30% when using reconstructions that include PSF for both repositioned and stationary data. Even in the stationary phantom study, recoveries varied with reconstruction protocol which is in agreement with Armstrong et al.^22^

### 4.B. Hoffman brain phantom

The Hoffman brain phantom consists of a complex structure that mimics the structure of the human brain. The measurement of tracer uptake in small brain structures such as the caudate and putamen can be hampered by partial volume effects. For the Philips Ingenuity system, the inclusion of the PSF in the reconstruction increased gray matter region RC_mean_ up to 5%–10% compared to those seen without PSF. On the other hand, RC_mean_ in white matter regions was reduced by 2%–5% when using PSF. These effects found for the Philips Ingenuity system are consistent with that by Shao et al.^23^ The data for the Siemens Biograph system were much less affected by use of PSF in the brain phantom experiment (< 2%), although visually images appear to have a higher resolution. These results can be expected as the use of PSF results in improved spatial resolution and should, therefore, result in higher recoveries in gray matter structures and lower ones for white matter. However, it should be noted that use of PSF may introduce Gibbs artifacts as well, which in turn could lead to activity concentration overestimations.^24^

Statistical analysis performed on the data from the Philips system showed a significant difference between repositioned and stationary phantoms scans for both gray and white matter VOIs. However, the differences were very small (< 5%) and likely not clinically relevant. The low sensitivity of RC variability for phantom repositioning likely results from the use of regionally averaged values. This was also observed in the NEMA IQ phantom, where SUV_mean_ seems to be less sensitive to phantom (re‐)positioning than SUV_max_. Therefore, spatially averaging data over an extended volume of interest seems to mitigate the effects of phantom repositioning and/or (voxel) sampling of the phantom. Although the distribution of the radiotracer in the Hoffman brain phantom is assumed to be uniform within gray and white matter regions, the distribution in a real human brain might exhibit larger variations. Therefore, it cannot be ruled out that there is an effect of patient repositioning on the precision of regional average values in clinical practice.

### 4.C. Future perspectives

This study confirms several findings from previous studies, such as precision dependence on scan statistics/duration, data analysis methods and reconstruction protocol, and may therefore be assumed to be generally applicable. In our work, we extended earlier studies by including the effects of repositioning in order to resemble the clinical conditions encountered in longitudinal studies for both oncology body scans as well as brain PET studies. We found that phantom repositioning and thereby tumor voxel sampling variations particularly affected the precision of SUV_max_ analysis for small spheres, while the use of regionally averaged values by SUV_peak_ or SUV_mean_ mitigated these uncertainties (in part). The latter can be understood easily as averaging data over multiple voxels mitigate some of the sampling effect. In particular, use of a fixed size VOI, such as SUV_peak_, generates uptake values that can be expected to be less influenced by voxel size provided fractional voxel coverage by the SUV_peak_ is taken into account appropriately, as was the case in this study.

A limitation of our work was the use of random repositioning of the phantom rather than applying systematic displacements in axial and transaxial directions. The latter would have allowed to determining the effect of axial versus transaxial resolution of the system on the observed precisions. In our study, we have chosen to randomly reposition the phantom to resemble clinical practice and we assumed that use of 12 or 10 replicates would provide sufficient understanding of PET uncertainty dependence on phantom repositioning as our results are in line with previous reports (using non‐PSF reconstructions^17^).

Secondly, in our paper, we focused only on some technical aspects or factors that could affect PET precision. Yet, there are many other sources of uncertainty in clinical practice,^25^ such as net injected activity, patient preparation procedures, uptake time variability, use of different data analysis software, scanner calibration errors, etc. that may have a much larger effect on PET precision than the effect of e.g., repositioning. The observed increased variability of SUV_max_ with IQ phantom repositioning is small compared to the uncertainties resulting from other factors, in particular when PET studies are not strictly performed in compliance with international guidelines. Yet, the authors believe that by using quantitative metrics, such as SUV_peak_, that may mitigate even relatively small sources of error could improve the repeatability and reproducibility of quantitative PET reads and are worth further exploration.

## 5 Conclusions

Precision of quantitative tracer uptake values depends on scan duration, data analysis methods, reconstruction protocol, and phantom repositioning. The latter effect was most pronounced in an oncological experimental phantom setting for smaller spheres (< 15 mm diameter) when using SUV_max_. When using either fixed sized VOIs (SUV_peak_ in the IQ phantom) or using regionally averaged data (brain phantom), the impact of phantom repositioning on quantitative precision is minimal. As in longitudinal studies it is impossible to exactly put the patient in the same position in the PET/CT system, it would be preferred to quantify tracer uptake using methods that are insensitive to patient repositioning. The use of SUV_peak_ in an oncological setting may, therefore, be a good alternative to SUV_max_, but its use for smaller lesions needs to be further studied due to the lower recoveries seen for spheres smaller than 15 mm diameter.

## Conflict of Interest

None. Dennis Heijtel is currently being employed by Philips Healthcare.

## Supporting information


**Fig. S1.** RC of NEMA IQ phantom data as a function of sphere diameter. Data acquired on the Philips Ingenuity system and based on images with a 4 × 4 × 4 mm^3^ voxel size and 2‐min starting frame duration using TOF on the left column and TOF + PSF on the right column. Figures (A and B) represent RC (%) for max, (C and D), peak, and (E and F) mean SUVs. Dotted lines correspond to the true RC based on the true activity within the phantom spheres. Boxes represent standard deviation (SD), whiskers show ranges, and solid line depicts median of the data.

**Fig. S2.** RC of NEMA IQ phantom data as a function of sphere diameter. Data acquired on the Siemens Biograph system and based on images with a 3.1819 × 3.1819 × 2 mm voxel size and 2‐min starting frame duration using TOF on the left column and TOF + PSF on the right column. Figures (A and B) represent RC (%) for max, (C and D), peak and (E and F) mean SUVs. Dotted lines correspond to the true RC based on the true activity within the phantom spheres. Boxes represent standard deviation (SD), whiskers show ranges, and solid line depicts median of the data.

**Fig. S3.** Maximum RC (%) of NEMA IQ phantom data as a function of sphere diameter. Data acquired on the Philips Ingenuity system and based on images with a 2 × 2 × 2 mm^3^ voxel size and 2‐min starting frame duration using TOF on the left and TOF + PSF on the right. Dotted lines correspond to the true RC based on the true activity within the phantom spheres. Boxes represent standard deviation (SD), whiskers show ranges, and solid line depicts median of the data.

**Fig. S4.** Maximum RC (%) of NEMA IQ phantom data as a function of sphere diameter. Data acquired on the Siemens Biograph system and based on images with a 2 × 2 × 2 mm^3^ voxel size and 2‐min starting frame duration using TOF on the left and TOF + PSF on the right. Dotted lines correspond to the true RC based on the true activity within the phantom spheres. Boxes represent standard deviation (SD), whiskers show ranges, and solid line depicts median of the data.
