# Supplementary figures and images for: Impact of PET/CT system, reconstruction protocol, data analysis method, and repositioning on PET/CT precision: An experimental evaluation using an oncology and brain phantom
Source: Med Phys. 2017 Nov 19;44(12):6413–24. doi: 10.1002/mp.12623 (PMC5734628; doi:10.1002/mp.12623)

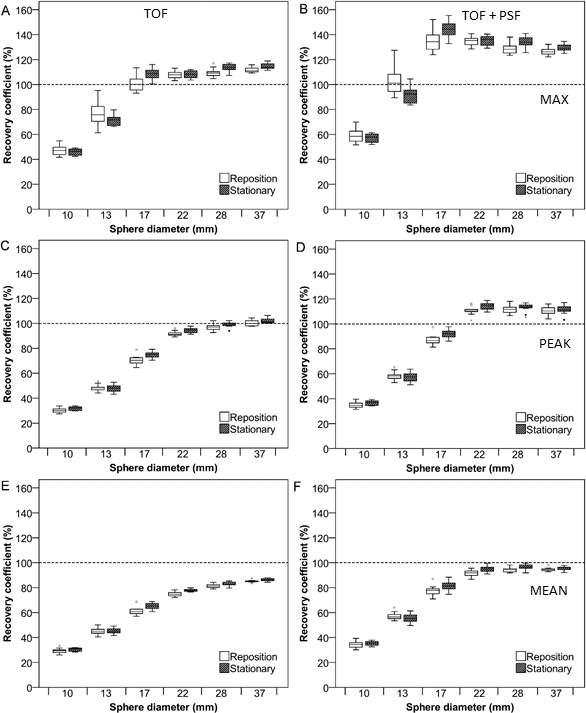

Supplement: Supplementary file 1 — Fig. S1. RC of NEMA IQ phantom data as a function of sphere diameter. Data acquired on the Philips Ingenuity system and based on images with a 4 × 4 × 4 mm3 voxel size and 2‐min starting frame duration using TOF on the left column and TOF + PSF on the right column. Figures (A and B) represent RC (%) for max, (C and D), peak, and (E and F) mean SUVs. Dotted lines correspond to the true RC based on the true activity within the phantom spheres. Boxes represent standard deviation (SD), whiskers show ranges, and solid line depicts median of the data. [file MP-44-6413-s001.tif]

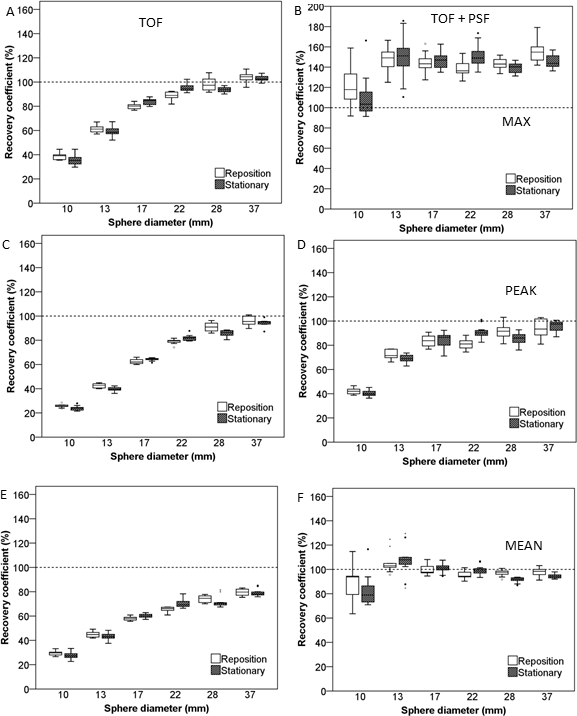

Supplement: Supplementary file 2 — Fig. S2. RC of NEMA IQ phantom data as a function of sphere diameter. Data acquired on the Siemens Biograph system and based on images with a 3.1819 × 3.1819 × 2 mm voxel size and 2‐min starting frame duration using TOF on the left column and TOF + PSF on the right column. Figures (A and B) represent RC (%) for max, (C and D), peak and (E and F) mean SUVs. Dotted lines correspond to the true RC based on the true activity within the phantom spheres. Boxes represent standard deviation (SD), whiskers show ranges, and solid line depicts median of the data. [file MP-44-6413-s002.tif]

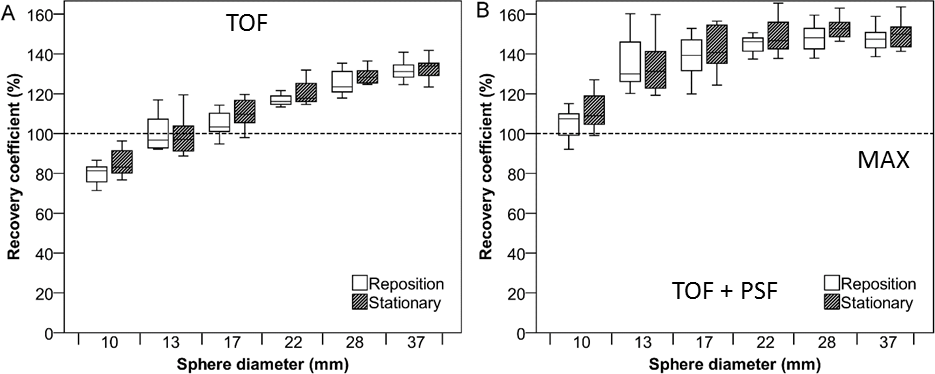

Supplement: Supplementary file 3 — Fig. S3. Maximum RC (%) of NEMA IQ phantom data as a function of sphere diameter. Data acquired on the Philips Ingenuity system and based on images with a 2 × 2 × 2 mm3 voxel size and 2‐min starting frame duration using TOF on the left and TOF + PSF on the right. Dotted lines correspond to the true RC based on the true activity within the phantom spheres. Boxes represent standard deviation (SD), whiskers show ranges, and solid line depicts median of the data. [file MP-44-6413-s003.tif]

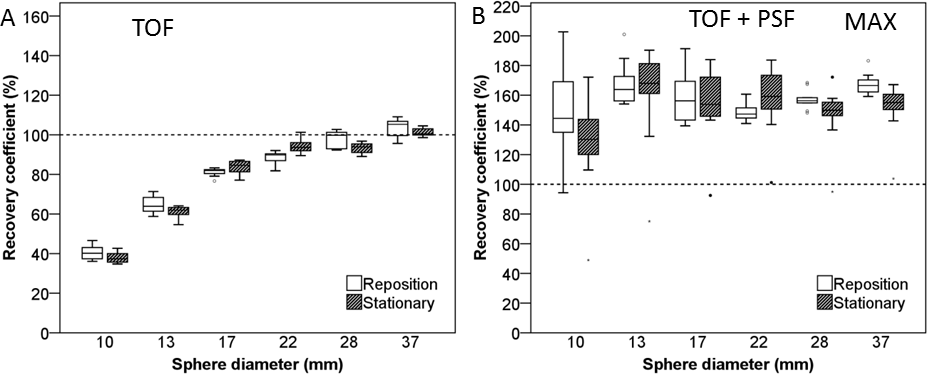

Supplement: Supplementary file 4 — Fig. S4. Maximum RC (%) of NEMA IQ phantom data as a function of sphere diameter. Data acquired on the Siemens Biograph system and based on images with a 2 × 2 × 2 mm3 voxel size and 2‐min starting frame duration using TOF on the left and TOF + PSF on the right. Dotted lines correspond to the true RC based on the true activity within the phantom spheres. Boxes represent standard deviation (SD), whiskers show ranges, and solid line depicts median of the data. [file MP-44-6413-s004.tif]
